# Oridonin Promotes Apoptosis and Restrains the Viability and Migration of Bladder Cancer by Impeding TRPM7 Expression via the ERK and AKT Signaling Pathways

**DOI:** 10.1155/2021/4340950

**Published:** 2021-07-05

**Authors:** Xianping Che, Jiangtao Zhan, Fan Zhao, Zunhe Zhong, Mianchuan Chen, Ruifa Han, Yi Wang

**Affiliations:** ^1^Department of Urology, The Second Affiliated Hospital of Hainan Medical University, 570311 Hainan, China; ^2^Department of Urology, The Second Hospital of Tianjin Medical University, Tianjin Institute of Urology, 300211 Tianjin, China

## Abstract

**Background:**

Oridonin is a powerful anticancer compound found in *Rabdosia rubescens*. However, its potential impact on bladder cancer remains uninvestigated. In this work, we aimed to detect the anticancer effect of oridonin on bladder cancer and explore the molecular mechanisms involved.

**Methods:**

The anticancer activity of oridonin was assessed in vitro with a CCK8 assay, an annexin V-FITC apoptosis analysis, and colony formation and Transwell migration assays which were performed with the human bladder cancer cell line T24. Levels of apoptosis-related proteins, melastatin transient receptor potential channel 7 (TRPM7), and signaling molecules were examined in oridonin-treated T24 cells by western blotting or RT-PCR. Oridonin anticancer efficacy was further validated in vivo with a T24 xenograft mouse model.

**Results:**

Oridonin repressed the proliferative, colony-forming, and migratory capacities of T24 cells, triggered extensive apoptosis in vitro, and retarded tumor growth in vivo. Moreover, oridonin treatment significantly increased expression levels of p53 and cleaved caspase-3 and reduced expression of TRPM7, p-AKT, and p-ERK.

**Conclusion:**

Oridonin exhibited outstanding antiproliferative and antimigratory effects on bladder cancer, and these effects were at least partially associated with targeting of TRPM7 through inactivation of the ERK and AKT signaling pathways. These findings provide insight for the clinical application of oridonin in bladder cancer prevention.

## 1. Introduction

Bladder cancer (BC) is reported to be the most frequent genitourinary malignant neoplasm in the three urological systems, and an estimated 81,400 new cases and 17,980 deaths are expected to occur in the United States in 2020 [1]. Non-muscle-invasive bladder cancer (NMIBC) is one of the common subtypes of BC and accounts for 75% of clinically diagnosed cases [2]. Although NMIBC is rarely fatal, its recurrence rate is higher than that of other types of BC [3]. The preferred approach for NMIBC therapy is surgical excision [4]. Currently, the instillation of chemotherapy or vaccine-based therapy after telescopic removal is performed to eradicate residual microtumors and diminish the risks of tumor recurrence and mortality [5–7]. However, the current method of chemotherapy or vaccine-based therapy instillation in BC has not been documented to be universally effective and fails to effectively treat 30-45% of NMIBC patients [8]. In addition, subsequent severe local or systemic complications inevitably arise [9]. Hence, more efficient therapeutic strategies for overcoming the bottleneck in the treatment of BC are strongly needed.

Transient receptor potential (TRP) channels are cation-selective ion channels on the cell membrane that serve as multifunctional sensors that respond to various stimuli, and they have been implicated in the pathological initiation and processes of cancer [10]. Melastatin transient receptor potential channel 7 (TRPM7), which is a TRP channel, contains an ion channel and a serine/threonine protein kinase and functionally controls Mg2+ and Ca2+ homeostasis [11]. Abnormal expression of TRPM7 is frequently observed in various cancers, including BC [12, 13]. These chanzymes, or channel kinases, can assist in epithelial-mesenchymal transition activation, facilitate cell migration and invasion, and promote cell survival during tumor transformation [14–16]. Furthermore, TRPM7 expression is positively correlated with several clinicopathological factors associated with the poor prognosis of several cancers [17, 18]; hence, this protein may represent a viable therapeutic target or biomarker for tumors.

More specifically, silencing TRPM7 enhances sensitivity to oxidative stress, increases the apoptosis-inducer BAX/BCL2 ratio, and contributes to the accumulation of cleaved caspase-3 [19]. It is also reported to influence cell motility and invasion by activating ERK1/2 [20], and previous reports have demonstrated that TRPM7 positively controls oncogenic AKT events to orchestrate the tumorigenesis, invasion, and metastasis of various cancers [14]. Therefore, targeted inhibition of TRPM7 expression might impede the initiation and progression of BC by downregulating ERK or AKT signaling pathway activity.

Currently, multiple pharmacological compounds and herbal remedies have been shown to effectively attenuate the proliferative and invasive capacities of tumor cells and thereby prevent malignancy via the blockade of TRPM7 channels [21]. One such example is waixenicin A, which is widely recognized as a potent inhibitor of TRPM7 and an efficient antitumor agent [22]. It is a natural terpenoid-related compound extracted from the soft coral *Sarcothelia edmondsoni*. Oridonin, a structurally analogous product of waixenicin A isolated from *Rabdosia rubescens*, has been documented to have a remarkable cancer-fighting and chemosensitization abilities for a broad spectrum of cancers mediated by regulating canonical oncogenic events [23, 24]. It can impact crucial intercellular functions, such as cell proliferation, migration, invasion, apoptosis, and autophagy, and thus produces deleterious effects on tumorigenic properties in cancers [25, 26]. However, its effects on BC have not been reported to date.

In this study, suppression of TRPM7 was hypothesized to promote the antitumor activity of oridonin via the AKT and ERK signaling pathways. Cell culture and animal studies were employed to test this hypothesis.

## 2. Materials and Methods

### 2.1. Chemicals and Reagents

Oridonin (99.85%) was obtained from MedChemExpress (Shanghai, China) and dissolved in a solution of 1% dimethyl sulfoxide (DMSO). Rabbit monoclonal antibodies against TRPM7, GAPDH, p-AKT, p-ERK, p53, and cleaved caspase-3 and horseradish peroxidase- (HRP-) conjugated secondary antibodies were purchased from Proteintech Group (Wuhan, China).

### 2.2. Cell Culture

Human T24 BC cells were obtained from the Shanghai Cell Bank of the Chinese Academy of Sciences and grown in DMEM supplemented with 10% FBS and 1% penicillin/streptomycin at 37°C with 5% CO_2_.

### 2.3. CCK8 Proliferation Assay

A commercial CCK8 assay kit (Dojindo, Shanghai, China) was used to measure the growth of T24 cells in different groups. In total, 2 × 10^3^ T24 cells were implanted in a 96-well plate and cultured until the cell confluence reached 75%. Then, the cells were treated with various concentrations of oridonin (0, 2, 4, 6, 10, 15, 20, and 30 *μ*M). After another 24 h incubation, the CCK8 assay solution (10 *μ*L) was added to every well and incubated for 4 h. The absorbance (OD value) of each well at 450 nm was examined with a microplate reader. The calculated mean values were utilized to construct a cell growth curve.

### 2.4. Migration Assay

The migratory capacities of different groups were appraised with Transwell migration assays. Briefly, 800 *μ*L of complete medium was placed into the lower chambers. Pretreated cells (2.5 × 10^3^) were seeded in the upper chambers with 200 *μ*L of serum-free medium containing 0, 1, 2, or 3 *μ*M oridonin. After culturing at 37°C for 24 h, the medium in the upper chambers was discarded and the cells were fixed with 4% formaldehyde, permeabilized with 100% methanol, and stained with crystal violet. Then, the stained cells were quantified under a light microscope.

### 2.5. Colony Formation Assay

To estimate the clonogenic activity in different treated groups, we performed a colony formation assay. Cells were grown in a 12-well plate (0.5 × 10^3^/well). After the cells had stably attached, the medium was discarded and the cells were continuously cultured in medium containing 0, 1, 2, or 3 *μ*M oridonin for 2 weeks. Next, images were acquired, and the number of visible colonies with ≥50 cells was recorded.

### 2.6. Apoptosis Assay

T24 cells (3 × 10^3^ cells/well) were grown in 6-well plates overnight and then incubated for 48 h with 0, 1, 2, or 3 *μ*M oridonin at 37°C. A total of 2 × 10^3^ cells were dispersed with trypsin and incubated with 2 *μ*L of annexin and 2 *μ*L of propidium iodide. After staining in the dark for 15 min, the samples were analyzed by flow cytometry and the apoptotic cell percentage was determined.

### 2.7. RT-qPCR

Total RNA was isolated from T24 cells treated with 0, 1, 2, or 3 *μ*M oridonin using TRI Reagent Solution (Ambion, Inc.). cDNA synthesis from 2 *μ*g of RNA was carried out with a RevertAid first-strand cDNA synthesis kit. The mRNA levels in T24 cells were quantified with SYBR Select Master Mix (2X) (ABI, USA). The relative mRNA levels of BAX and FOXO3 were analyzed by the 2^−ΔΔCt^ method.

The following primers were used: GAPDH: forward, 5′-CATGGCACCGTCAAGGCTGA-3′, and reverse, 5′-ACGTACTCAGCGCCAGCATC-3′, and FOXO3: forward, 5′-CCGCTGTGTCTGCCCAGAAT-3′, and reverse, 5′-GTGCTGGTGGTGGAGCAAGT-3′.

### 2.8. Western Blotting

A western blot analysis was performed according to a traditional method. In brief, total protein was isolated from T24 cells treated with different oridonin concentrations using the alkaline lysis method. The protein concentration was measured with a bicinchoninic acid (BCA) protein assay kit. Twenty micrograms of protein was loaded on 10% SDS-PAGE gels, electrophoresed at a constant voltage of 80 volts, and electrotransferred to PVDF membranes. Nonfat milk (5%) was used to prevent nonspecific binding on the membranes. Subsequently, the membranes were incubated with primary antibodies and then secondary antibodies. The bands were visualized with chemiluminescence, scanned with a phosphor imager, and then quantified with ImageJ. The primary antibodies employed in this study were rabbit monoclonal antibodies against TRPM7, GAPDH, p-AKT, p-ERK, P53, and cleaved caspase-3.

### 2.9. Construction of a TRPM7 Overexpression Vector and Infection

TRPM7 cDNA was amplified from T24 cells and inserted into the pCMVp-NEO-X vector using TRPM7-specific primers. Subconfluent HEK293T cells were transfected with the produced plasmid pCMVp-NEO-TRPM7 using the calcium-phosphate method. After 48 h, the lentiviral particles were collected and filtered and used to transfect the subconfluent T24 cells for 72 h. Stably transfected cells were subjected to 8 days of selection with 400 *μ*g/mL of G418. After confirmation by western blotting, the surviving cells were treated with or without 3 *μ*M oridonin for 24 h and prepared for subsequent experiments.

### 2.10. In Vivo Antitumor Effect of Oridonin on Bladder Cancer

The animal studies were authorized by the Ethics Committee of the Second Affiliated Hospital of Hainan Medical College (China) and performed in line with the principles of the NIH Guide for the Care and Use of Laboratory Animals.

Fifteen C57BL/6 female mice (SPF, 7 weeks old) were purchased from the Experimental Animal Center of Three Gorges University (Yichang, China) and housed in a sterile environment. Subcutaneous injection of 5 × 10^6^ T24 cells was performed to establish xenograft models of BC. When the tumor volumes reached 200 mm^3^, the animals were separated into 3 groups (*n* = 5) and injected with 1% normal saline (control group), 5 mg/kg/d oridonin, or 10 mg/kg/d oridonin. Tumor size (length × width^2^/2) and body weight were measured every other day until posttreatment day 21. At the end of the experiments, all animals were euthanized under urethane anesthesia and tumor tissues were obtained from each mouse.

### 2.11. Statistical Analysis

Statistical comparisons between the control group and treatment groups were performed via unpaired Student's *t*-test or ANOVA. Statistical significance was set at *P* < 0.05. The analyses were performed using Prism 8.

## 3. Results

Oridonin inhibited T24 cell proliferation, migration, and colony formation in vitro.

**Figure 1:**
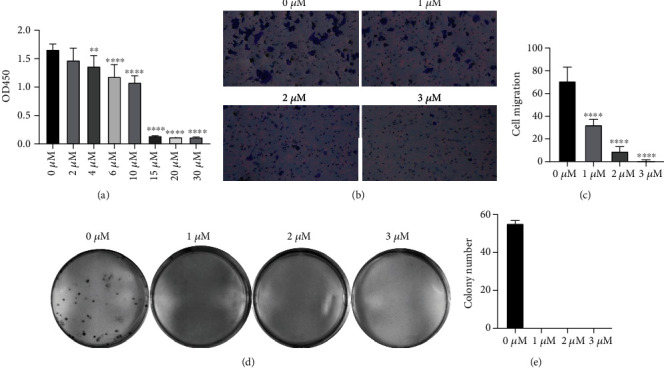
Oridonin limited T24 cell proliferation, colony formation, and migration. (a) Proliferation of T24 cells treated with oridonin (0, 2, 4, 6, 10, 15, 20, or 30 *μ*M). (b, c) Migratory ability of T24 cells treated with oridonin (0, 1, 2, or 3 *μ*M) and vs. the untreated control (0 *μ*M). Magnification ×200. (d, e) Colony formation capacity of T24 cells treated with oridonin (0, 1, 2, or 3 *μ*M).

CCK8 assays were first adopted to clarify the effect of oridonin on BC in vitro. As shown in Figure 1(a), the proliferation rate of T24 cells was significantly attenuated after 24 h of exposure to different doses of oridonin. The proliferative capacity was impaired to 62.70% and 0.99% when the cells were treated with 10 *μ*M or 30 *μ*M oridonin, respectively. Compared with the oridonin treatment group, however, the control group showed no impairment in the proliferation rate. Transwell migration assays were performed to evaluate the effects of oridonin on the migratory activity of T24 cells. At 24 h posttreatment with oridonin, the migrated cell number was reduced in a dose-dependent manner (Figures 1(b) and 1(c)). Unsurprisingly, a significant decrease in the colony formation rate of T24 cells was also observed after oridonin treatment. All findings are suggestive of the antitumor potential of oridonin.

### 3.1. Oridonin Induces Apoptosis in T24 Cells

**Figure 2:**
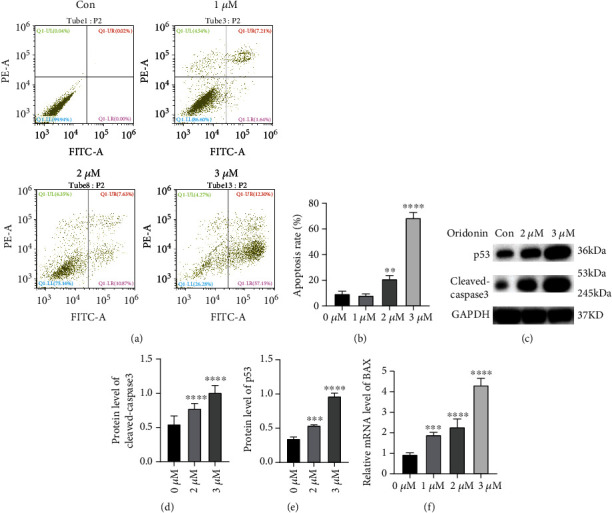
Dose-dependent induction of apoptosis in T24 cells by oridonin. (a, b) Annexin V-FITC- and PI-stained cells were quantified by flow cytometry after the T24 cells were incubated with oridonin (0, 1, 2, or 3 *μ*M). (c–e) p53 and cleaved caspase-3 protein levels in T24 cells were assessed after the cells were incubated with oridonin (0, 1, 2, or 3 *μ*M). (f) BAX mRNA levels in T24 cells incubated with oridonin (0, 1, 2, or 3 *μ*M) were determined. ^∗∗∗^*P* < 0.001 and ^∗∗∗∗^*P* < 0.0001 vs. the untreated control (0 *μ*M).

Oridonin-activated proapoptotic activity is well characterized in several cancer cell lines; therefore, annexin V/PI staining was implemented to verify whether oridonin treatment can induce apoptosis in T24 cells. The results demonstrated that apoptotic events were successfully triggered in a dose-dependent manner via the oridonin treatment (Figures 2(a) and 2(b)). In particular, oridonin administration considerably increased late apoptosis but showed a marginal impact on early apoptosis (Figure 2(a)). Further characterization was performed to examine the expression levels of apoptosis-related factors in T24 cells after oridonin treatment. The p53 and cleaved caspase-3 protein levels and BAX mRNA level were higher in the T24 cells exposed to oridonin than in the control cells, and expression of these molecules increased in a concentration-dependent manner (Figures 2(c)–2(f)). These results indicated that oridonin potently triggered apoptosis in T24 cells.

### 3.2. Oridonin Inactivates AKT and ERK Signaling Pathways by Inhibiting TRPM7 Expression

**Figure 3:**
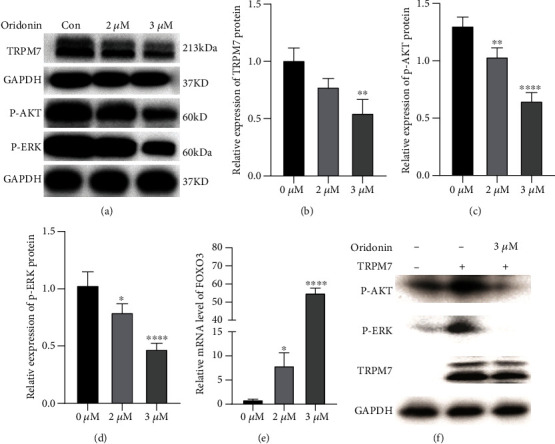
Oridonin inactivates the AKT and ERK signaling pathways by inhibiting TRPM7 expression. (a–d) Western blotting was performed to determine the expression of TRPM7, p-AKT, and p-ERK after the T24 cells were treated with 0, 2, and 3 *μ*M oridonin. (b–e) RT-PCR was performed to determine the mRNA expression of FOXO3 after T24 cells were treated with 0, 2, and 3 *μ*M oridonin. (f) T24 cells were infected with TRPM7-overexpressing plasmids, and then, TRPM7, p-AKT, and p-ERK expression was examined after treatment with and without 3 *μ*M oridonin. ^∗^*P* < 0.05, ^∗∗^*P* < 0.01, ^∗∗∗^*P* < 0.001, and ^∗∗∗∗^*P* < 0.0001 vs. the untreated control (0 *μ*M).

Since TRPM7 is a key checkpoint and regulator of the activity of some terpenoids against cancer [22, 27], we also examined the TRPM7 expression level upon oridonin treatment. Figures 3(a) and 3(b) demonstrate that the TRPM7 protein level progressively decreased with increases in the oridonin dose. TRPM7 inhibits the AKT and ERK pathways to promote malignancy in cancers [28, 29]. Therefore, the critical propagators p-AKT and p-ERK in the aforementioned two signaling pathways were assessed upon oridonin treatment. An immunoblot assay demonstrated that compared with the control T24 cells, the oridonin-treated T24 cells displayed a considerable decrease in the protein levels of p-AKT and p-ERK as the concentration of oridonin increased (Figures 3(c) and 3(d)). We also evaluated the FOXO3 mRNA level since FOXO3 is reported to be a critical downstream molecule that negatively impacts oncogenic PI3K/Akt events. As expected, the FOXO3 mRNA level increased as the concentration of oridonin increased (Figure 3(e)).

To further elucidate the role of TRPM7/AKT and ERK in oridonin antitumor activity, western blot assays were performed to detect p-AKT and p-ERK expression after TRPM7-overexpressing T24 cells were treated with 3 *μ*M oridonin. The results showed that the oridonin treatment could offset the increased p-AKT and p-ERK expression levels induced by highly expressed TRPM7 in T24 cells (Figure 3(f)). These findings suggest that oridonin inhibits the AKT and ERK signaling pathways by inhibiting TRPM7 expression, which results in the suppression of the malignant behavior of T24 cells.

### 3.3. Oridonin Suppresses Tumor Growth in a T24 Xenograft Tumor Model

**Figure 4:**
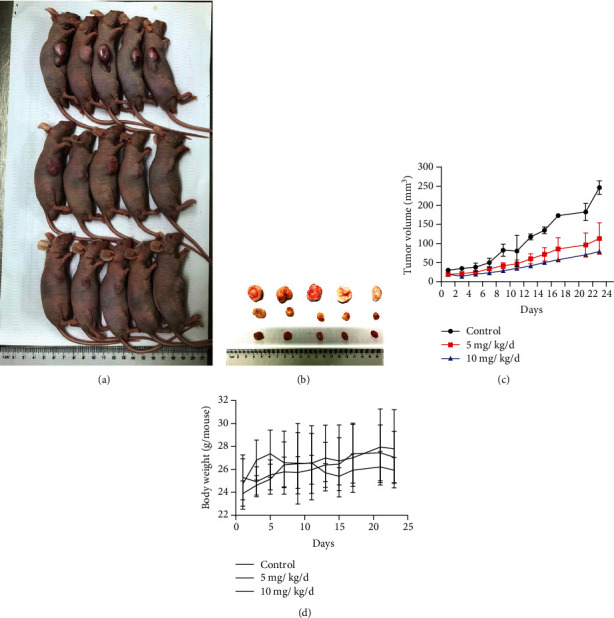
In vivo effect of oridonin on tumorigenicity in T24 xenograft tumor model mice. (a) Representative images of T24 tumor-bearing mice given different treatments for 23 days. (b) Flank tumors resected from mice bearing T24 xenograft tumors. (c) Tumor volume curves of T24 xenograft model mice under different treatments (*n* = 5). (d) Tumor weight curve of T24 xenograft model mice under different treatments (*n* = 5).

Considering the antineoplastic activity of oridonin observed in vitro, we further investigated its curative potential in a successfully established T24 xenograft tumor model (Figure 4(a)). Our data demonstrated that intraperitoneal injection of oridonin significantly restrained tumor growth as evidenced by the obviously decreased tumor volumes in the treated groups compared with those in the control group (Figure 4(b)). The 10 mg/kg/d group showed enhanced suppression of tumor growth (Figure 4(c)); however, no statistically significant changes in body weight were observed among these three groups (Figure 4(d)). These findings reveal that oridonin has inhibitory potential against BC in vivo.

## 4. Discussion

Although rational therapy for BC has produced encouraging prognoses in BC patients, postoperative metastasis and recurrence continue to be the main challenges in BC treatment [30]. Hence, alternative therapeutic interventions are strongly needed to treat BC progression. Here, we evaluate the antineoplastic activity of oridonin in BC in vitro and in vivo and explore the underlying mechanism. The results showed that oridonin significantly impaired T24 cell proliferation, colony formation, and migration and induced apoptosis by suppressing TRPM7 expression, which regulated the ERK and AKT signaling cascades.

Recently, a myriad of investigations have shown that oridonin can effectively retard tumor growth in various kinds of cancers [31]. However, previous investigations have not focused on the impact of oridonin on BC. Our work is the first report to address the antiproliferative activity of oridonin in the BC cell line T24, and the results revealed that oridonin administration can significantly constrain the proliferative capacity of this BC cell line. The antiproliferative effects of oridonin on cancer cells were associated with apoptosis induction, which is a critical indication of one of the therapeutic effects of antitumor agents. Therefore, the apoptotic rates of T24 cells upon oridonin exposure were assayed. Oridonin-treated T24 cells displayed strong apoptosis, and at concentrations up to 1 *μ*M, the percentage of apoptotic T24 cells was increased to 68.62 ± 2.306%. p53 is a well-established apoptosis inducer, and its activation can result in increased expression of proapoptotic factors and reduced expression of antiapoptotic genes [32]. Cleaved caspase-3 protein upregulation has a critical function in intracellular protein lysis and the characteristic alterations in morphology indicative of apoptosis [33]. Therefore, western blotting was performed to detect apoptosis-related protein expression, and p53 and cleaved caspase-3 expression levels increased as the concentration of oridonin increased, revealing that oridonin triggers apoptosis in T24 BC cells. These results suggest that oridonin exerts antiproliferative effects on T24 cells to retard tumor growth by inducing apoptosis. The findings were confirmed by the fact that oridonin constrained T24 tumor growth in nude mice.

TRPM7 was previously discovered to be a potent driver of oncogenic transformation through collaboration with MMP2 and cyclin D1 and is closely associated with the hallmark capabilities of cancers, such as sustained chronic proliferation, evasion of growth suppressors, and activation of invasion and metastasis [34–36]. TRPM7 is constitutively activated in various kinds of tumors, including BC [37]. Moreover, upregulation of TRPM7 expression serves as a powerful indicator of clinicopathological features related to relatively poor clinical outcomes for individuals with various types of cancers [37]. TRPM7 silencing can potentially limit the proliferative, invasive, and migratory capacities of BC. Thus, inactivation of this ion channel is considered a potential therapeutic modality for cancer treatment and has garnered substantial interest. Waixenicin A, a natural terpenoid, has been identified as an efficient suppresser of TRPM7 and shows deleterious effects on the malignant behaviors of human gastric and breast adenocarcinoma cells [34]. Oridonin has been reported as a terpenoid that shares a similar functional structure with waixenicin A [38]. Therefore, TRPM7 expression was examined in T24 cells treated with oridonin. As expected, we found that oridonin effectively reduced expression of TRPM7, which is related to the antiapoptotic, proliferative, adhesive, and invasive potential of BC. Previous studies have demonstrated that the inhibition of TRPM7 inactivates ERK or AKT signaling events and thereby influences various cancer cellular fates [14, 20]. Moreover, reports have shown that oridonin lessens the malignant phenotypes of transformed cells by inactivating ERK and AKT signaling events in lung cancer [39]. Hence, we further measured the phosphorylation level of ERK to elucidate the potential regulatory mechanisms of oridonin. After treatment with oridonin, T24 cells had reduced levels of phosphorylated ERK and AKT. These findings demonstrated that oridonin might inhibit T24 cell viability and migration by weakening TRPM7 expression via regulation of the ERK and AKT signaling cascades.

In conclusion, oridonin has therapeutic anticancer potential and functions by targeting TRPM7 via inactivation of ERK and AKT signaling. Thus, oridonin may be a safe pharmacological intervention for preventing BC.

## Data Availability

The data used to support the findings of this study are included within the article.
